# The association of long-term calcium and dairy products intake in adolescence with carotid intima media thickness and metabolic syndrome in early adulthood: Tehran Lipid and Glucose Study

**DOI:** 10.1186/s12986-023-00725-4

**Published:** 2023-04-03

**Authors:** Assa AkbarySedigh, Golaleh Asghari, Maryam Mahdavi, Parvin Mirmiran, Majid Valizadeh, Fereidoun Azizi

**Affiliations:** 1 Nutrition and Endocrine Research Center, Research Institute for Endocrine Sciences, Shahid Beheshti University of Medical Sciences, P.O. Box: 19395-4763, Tehran, Iran; 2 Department of Clinical Nutrition and Dietetics, Faculty of Nutrition Sciences and Food Technology, Shahid Beheshti University of Medical Sciences, Tehran, Iran; 3 Obesity Research Center, Research Institute for Endocrine Sciences, Shahid Beheshti University of Medical Sciences, P.O. Box: 19395-4763, Tehran, Iran; 4 Endocrine Research Center, Research Institute for Endocrine Sciences, Shahid Beheshti University of Medical Sciences, Tehran, Iran

**Keywords:** Calcium, Dairy, cIMT, Metabolic syndrome, Adolescence, Early adulthood

## Abstract

**Background:**

Calcium could impact on vascular functions and structures and cause atherosclerosis. Thus, we aimed to examine the association of long-term calcium and dairy products intake in adolescence with cIMT and MetS in early adulthood.

**Methods:**

We considered 217 adolescents aged 12–18 years in the frame work of the Tehran Lipid and Glucose Study (2006–2009) and follow-up them to early adulthood (2015–2017). The valid food frequency questionnaire was used to assess dietary intake. Ultrasound examination was used to measure common carotid artery. The joint interim statement and cook et al. criteria were used for adults and adolescents to consider MetS, respectively.

**Results:**

Adolescents’ average calcium intake from dairy and non-dairy sources were 395 mg/d and 1088 mg/d, respectively while adults had 212 mg/d and 1191 mg/d. In addition, the mean of cIMT in adults was 0.54 mm. There was no relationship of non-dairy (β: − 0.03; *P* = 0.804), and total calcium (β: − 0.001; *P* = 0.591) intake with cIMT and TG. None of the dairy products had link with cIMT, MetS and its components, except cream with cIMT after full adjustment of potential confounders (β: 0.245; *P* = 0.009). Also, we found that the intake of non-dairy products could increase DBP after controlling for potential confounders (β: 0.365; *P* = 0.012). Adolescence with higher quartiles of total calcium intake had no odds ratio of MetS in early adulthood (β: 2.05, *P* = 0.371).

**Conclusions:**

Adolescence calcium and dairy products intake, with the exception of cream did not increase early adulthood cIMT and MetS and its components.

**Supplementary Information:**

The online version contains supplementary material available at 10.1186/s12986-023-00725-4.

## Introduction

Cardiovascular disease (CVD) which is one of the major concerns and causes of death all over the world in the last few decades, develops in a prolonged period of time, and stems from unhealthy dietary patterns as well as sedentary lifestyle [1]. Also, it could happen after increasing of carotid intima media thickness (cIMT), metabolic syndrome, and atherosclerosis [2–4]. Hence, the early diagnosis of these triggers in addition to modifications of food habits are the prominent approach to prevent the chronic and dangerous symptoms and CVD.

Eating habits and nutrients have an effective role in incidence of metabolic syndrome and atherosclerosis in a long-life time [5–9]. Particularly, calcium has been claimed to impact on vascular functions and structures in the way to contract myocardium, the muscle of heart and also coagulate blood [10]. Needless to say that dairy products providing calcium requirements are not exceptional. In a double-blind randomized trial study, women were given calcium supplementation with 2-year duration and the results showed that there was a significant increment in cholesterol levels and cIMT in postmenopausal women [11]. whereas, long-term dietary calcium intake from childhood to adulthood was not associated with any cardiovascular risk including cIMT, arterial pulse wave velocity (PWV), and stiffness index [12]. In terms of dairy products, some review studies concluded that CVD could be positively or impartially influenced by dairy intake, especially yoghurt, kefir and cheese [13–15]. However, some evidence claimed that high consumption of calcium might have detrimental effects on CVD [16–18]. Therefore, there are some controversies and inconsistency of findings needing to become more clear by further research and studies.

To the best of our knowledge, the long-term relationship between calcium and dairy products with CVD risks from childhood and adolescence to young adulthood has not been examined yet. Thus, the present study was conducted to clarify if there is a negative or positive impact on vascular structure and metabolic syndrome by calcium intake from dairy and non-dairy after eight years.

## Methods and materials

### Population of the study

The present study has been conducted in the frame work of Tehran Lipid and Glucose Study (TLGS) in the district 13 of Tehran since 1999. The details have been previously released [19]. The participants in the cohort study were followed up every three years. The cohort study has six phases that we considered the third phase (2006–2009) for adolescents and the sixth phase (2015–2017) for adults. The following flowchart shows all inclusion and exclusion criteria in details: first, we considered adolescents aged 12–19 years from the third phase (n = 1546) and adults aged over 20 years from the sixth phase (n = 1024). The number of adolescents filled the FFQ in the third phase were only 274. Then, we excluded adolescents having over- and under-reported energy intake (n = 1), having diseases such as high blood pressure, hyperglycemia, and high lipid profile (n = 23), underweight (n = 33). Lastly, the number of participants considered for final analysis were 217. The flowchart was presented in the Additional file 1. Regarding selection bias, we compared all healthy adolescents having cIMT measurements in the sixth phase with those 217 adolescents included in the current study. The results showed that our participants were more likely to be younger (*P* < 0.001) and non-smokers (*P* = 0.007). All other variables were the same (data not shown)*.* Noteworthy, the reason that we did not consider the first and second phases was that the nutrition data (FFQ) have been started collecting from the third phase. Also, cIMT measurements have been carried out from the sixth phase. All participants were given to sign the written consent to take a part. The ethics research council of the Research Institute for Endocrine Sciences, Shahid Beheshti University of Medical Sciences consented the protocol of this study.

### Dietary intake and physical activity assessment

The valid and reliable FFQ was used to assess dietary intakes [20]. The participants were asked about 168 food items and the consumption frequency over the past year on a daily, weekly, and monthly basis during face-to-face interview. Household measures were taken into account for portion sizes and then converted to grams. The food composition table (FCT) of the United States Department of Agriculture (USDA) was used to evaluate energy and nutrients. The Iranian FCT was considered for local foods that were not existed in USDA FCT. In addition, both adolescents and adults answered questions about their leisure and daily activities by Modifiable Activity Questionnaire (MAQ) and Lipid Research Clinics (LRC) questionnaire [21, 22].

### Anthropometric and clinical measurements

Participants were interviewed to collect information about demographic, medical history and smoking status. Height and weight were assessed by a digital scale and an elastic tape with light clothing and without shoes in the standard position. Waist circumference (WC) was recorded at the umbilicus [23]. Body Mass index (BMI) was calculated as weight (Kg) divided by the square of height (m^2^).

After 12–14 h overnight fasting, blood samples were drawn into vacutainer tubes to measure total cholesterol, triglyceride, high-density lipoprotein (HDL), low-density lipoprotein (LDL), fasting blood sugar (FBS) between 7:00 and 9:00 am. Moreover, their systolic and diastolic blood pressure (SBP, DBP) were measured by a mercury sphygmomanometer and Korotkoff sound technique by an experienced physician after 15 min resting.

### Carotid intima-media thickness

Ultrasound examination of the subjects was done with a linear 7.5–10 MHz transducer (Samsung Medison SonoAceR3 ultrasound machine). Participants in the supine position with the neck extended and slightly rotated to the opposite side of examination. The measurements were performed on both left and right carotids. The decision on using the left common carotid artery (LCCA) was based on previous published articles in the literature [24]. Initial carotid scan was performed in the transverse plane throughout the course of the artery to evaluate the subject’s anatomy, locate atherosclerotic plaques (if there were any) and determine the site of maximal wall thickening in the near or far wall. Then longitudinal scans of the artery with different angles were performed. Measurements were done in plaque-free arterial segments which also fulfilled the criteria of optimal B-mode imaging as described below. cIMT was done manually as a hypo echoic band between the echogenic intimal and adventitial surfaces of the arterial wall. The distance between the leading edge of the first and second echogenic lines of the far walls of the distal segment of the common carotid artery on both sides was measured in three locations with the average as the final measurement of that side. The cIMT of carotid bulb and the internal carotid artery on both sides were sporadically measured in patients that fulfilled the criteria of optimal technique and image. To assess the reliability agreement, cIMT was measured by two radiologists in a subsample of 30 individuals (66.7% female) with mean age and BMI of 41.7 years and 24.4 kg/m^2^, respectively. The degree of cIMT measures agreement between the two radiologists was evaluated by using an interclass correlation coefficient (ICC). ICC estimates and the 95% confident intervals were calculated using SPSS version 20 based on 2-way mixed-effects model and reported ICC results as ICC = 0.79 with 95% confident interval = 0.55–0.90. The ICC is a value between 0 and 1, where values between 0.75 and 0.9 indicates good reliability [25].

### Metabolic syndrome

For adolescents, we used the definition provided by cook et al. due to no universal definition exists [26]. Metabolic syndrome (MetS) has been defined as 3 or more of the following factors: fasting triglyceride (TG) ≥ 110 mg/dL, HDL-C < 40 mg/dL, WC ≥ 90 percentile for age and sex, based on national reference curves [27], systolic blood pressure and/or diastolic blood pressure ≥ 90 percentile for age, sex, and height, in terms of National Heart, Lung, and Blood Institute’s recommended cut-off points [28], and FBS ≥ 100 mg/dL, as stated by the recent recommendation of American Diabetes Association [29]. Regarding the joint interim statement (JIS) criteria in adults, it defines MetS as ≥ 3 features including [30]: WC ≥ 91 cm for women and ≥ 89 cm for men based on population- and country- specific cut- off point for Iranians [31], fasting plasma glucose ≥ 100 mg/dL or drug treatment, TG ≥ 150 mg/dL or drug treatment, HDL-C < 50 mg/dL for women and < 40 mg/dL for men or drug treatment, and escalated blood pressure was defined as systolic blood pressure ≥ 130 mm Hg, diastolic blood pressure ≥ 85 mm Hg or antihypertensive drug treatment.

### Statistical analysis

Kolmograph—Smirnov test and histogram was used to determine the normal distribution of the data. Continuous and categorical variables reported by mean ± SD for normal and median (25th–75th percentiles) for skewed variables. Differences in anthropometric and clinical characteristics in adolescence and adulthood were tested using the paired t-test, Wilcoxon and McNemar test for normal, skewed, and categorical variables, respectively. Linear regression was used to investigate the association of adolescence dairy, non-dairy, and total calcium intake with cIMT, and MetS components in early adulthood. Binary logistic regression was used to examine the MetS of adulthood across quartiles of calcium and dairy intake in adolescence. We also considered potential confounding factors such as age, sex, BMI, physical activity, energy intake, smoking and family history of CVD in all analysis. In the current study, SPSS software version 20 (Chicago, IL, United State) was used for data analysis. *P*-value < 0.05 was considered statistically significant.

## Results

In the current study, as Table 1 shows the anthropometric and clinical measurements in adolescence and adulthood, the mean age of adolescents and adults were 14.9 years old and 24.9 years old, respectively. Also, the averages of BMI were 23.1 kg/m^2^ and 25.6 kg/m^2^ in the adolescents and adults. Adolescents’ average calcium intake from sources of dairy and non-dairy were 395 mg/d and 1088 mg/d while adults had 212 mg/d and 1191 mg/d. In addition, the mean of cIMT in adults was 0.54 mm. The number of adolescents and adults with MetS were 27 and 22, respectively. All components of MetS had difference between adolescence and adulthood (*P* < 0.001) except TG (P: 805) as well as non-dairy intake (P: 0.060). The mean of total calcium intake decreased from adolescence (1484 mg/d) to adulthood (1403 mg/d) while, the mean of HDL-C, SBP and DBP, and WC increased after 10 years (*P* < 0.001).

**Table 1:** Anthropometric and clinical measurements in adolescence and adulthood (n = 217)

Variables	Adolescence	Adulthood	*P*-value
Age (year)	14.9 ± 2.1	24.9 ± 2.9	< 0.001
BMI (kg/m^2^)	23.1 ± 4.3	25.6 ± 4.7	< 0.001
Dairy calcium (mg/d)	395 ± 573	212 ± 165	< 0.001
Non-dairy calcium (mg/d)	1088 ± 574	1191 ± 473	0.060
Total calcium (mg/d)	1484 ± 982	1403 ± 568	0.337
Energy intake (Kcal/d)	2693 ± 1087	2440 ± 871	0.005
Physical activity (MET/h)	536 (259–1488)	496 (246–1127)	0.035
^a^Smokers, n (%)	50 (23.0)	32 (14.7)	0.127
LDL-C (mgl/dl)	86.6 ± 23.5	96.1 ± 26.2	< 0.001
Cholesterol (mg/dl)	152.9 ± 28.4	165.9 ± 31.5	< 0.001
HDL-C (mgl/dl)	45.3 ± 10.0	48.7 ± 10.3	< 0.001
TG (mg/dl)	86.5 (65.0–120.0)	86.5 (66.2–126.0)	0.805
FBS (mg/dl)	87.6 ± 8.5	87.4 ± 7.9	0.004
SBP (mmHg)	102.2 ± 11.0	107.0 ± 11.4	< 0.001
DBP (mmHg)	66.4 ± 9.3	71.8 ± 8.9	< 0.001
WC (cm)	78.4 ± 11.9	86. 8 ± 12.2	< 0.001
cIMT (mm)	–	0.54	–
MetS, n (%)	27 (12.4)	22 (10.1)	> 0.999

Using the paired t-test, Wilcoxon and McNemar test for normal, skewed, and categorical variables, respectively

*P*-value < 0.05 significant

BMI, body mass index; cIMT, carotid intima media thickness; DBP, diastolic blood pressure; FBS, fasting blood sugar; HDL-C, high density lipoprotein-cholesterol; LDL, low density lipoprotein-cholesterol; MetS, metabolic syndrome; SBP, systolic blood pressure; TG, triglyceride; WC, waist circumference

^a^Definition of smokers for adolescent was both exposure and using smoke

Table 2 presents the associations of adolescence dairy, non-dairy, and total calcium intake with cIMT, and MetS components in early adulthood. There were no relationships of dairy (β: 0.041; 95% CI 0.000, 0.000; P: 0.647), non-dairy (β: 0.006; 95% CI 0.000, 0.000; P: 0.946) and total calcium (β: 0.029; 95% CI 0.000, 0.000; P: 0.752) intake with TG after controlling for age, sex, BMI, and physical activity. While, after further adjusting energy intake, smoking, and family history of cardiovascular disease, we observed the associations of non-dairy (β: 0.274; 95% CI 0.000, 0.000; P: 0.046) and total calcium intake (β: 0.275; 95% CI 0.000, 0.000; P: 0.039) with TG but not with dairy intake (β: 0.133; 95% CI 0.000, 0.000; P: 0.205). Moreover, we found that the intake of non-dairy calcium could increase DBP after adjusting for the potential confounders (β: 0.365; 95% CI 0.001, 0.010; P: 0.012). However, there were not any association of dairy, non-dairy, and total calcium intake with cIMT and other components of MetS including HDL-C, FBS, SBP, and WC.

**Table 2:** Associations of adolescence dairy, non-dairy, and total calcium intake with cIMT, and MetS components in early adulthood. (n = 217)

Outcomes	Non-dairy calcium		Dairy calcium		Total calcium	
	SC (β)	*P*-value	SC (β)	*P*-value	SC (β)	*P*-value
*cIMT (mm)*						
Model 1	− 0.03	0.800	− 0.07	0.477	− 0.001	0.988
Model 2	− 0.03	0.804	− 0.05	0.607	− 0.07	0.591
*HDL-C (mmol/l)*						
Model 1	− 0.151	0.078	− 0.086	0.350	− 0.142	0.119
Model 2	− 0.017	0.901	0.034	0.740	0.025	0.848
*TG (mmol/l)*						
Model 1	0.006	0.946	0.041	0.647	0.029	0.752
Model 2	0.274	0.046	0.133	0.205	0.275	0.039
*FBS (mmol/l)*						
Model 1	0.148	0.108	0.104	0.268	0.148	0.114
Model 2	0.177	0.212	0.075	0.487	0.167	0.226
*SBP (mmHg)*						
Model 1	− 0.022	0.809	− 0.162	0.078	− 0.111	0.230
Model 2	0.084	0.552	− 0.147	0.170	− 0.103	0.456
*DBP (mmHg)*						
Model 1	0.212	0.022	0.091	0.342	0.177	0.063
Model 2	0.365	0.012	0.076	0.499	0.266	0.062
*Waist circumference (cm)*						
Model 1	0.104	0.130	0.073	0.296	0.104	0.136
Model 2	0.152	0.148	0.044	0.584	0.123	0.229

Model 1. Adjusted for age, sex, body mass index, and physical activity

Model 2. Adjusted for Model 1 and energy intake, smoking, family history of cardiovascular disease

Using Linear regression test

*P*-value < 0.05 significant

cIMT, carotid intima media thickness; DBP, diastolic blood pressure; FBS, fasting blood sugar; HDL-C, high density lipoprotein-cholesterol; SBP, systolic blood pressure; SC, standardized coefficients; TG, triglyceride

Table 3 indicates the associations of adolescence dairy and non-dairy calcium intake with odds ratio of MetS in early adulthood. We found that adolescents with higher quartiles of calcium intake had higher odds ratio of MetS in early adulthood in comparison with first quartile after controlling for potential confounders; however, it was not statistically significant (OR 2.05; 95% CI 0.12, 35.17; P: 0.371).

**Table 3:** Associations of adolescence total calcium intake with odds ratio of MetS in early adulthood (n = 217)

Outcomes	Q1	Q2	Q3	Q4	*P*-value
*MetS*					
Model 1	1	1.81 (0.26–12.46)	2.87 (0.50–16.54)	1.79 (0.25–12.66)	0.443
Model 2	1	1.35 (0.16–10.99)	2.70 (0.38–18.84)	2.05 (0.12–35.17)	0.371

Model 1. Adjusted for age, sex, body mass index, and physical activity

Model 2. Adjusted for Model 1 and energy intake, smoking, family history of cardiovascular disease

Using Binary logistic regression test

*P*-value < 0.05 significant

MetS, metabolic Syndrome

Noteworthy, as Additional file 2: Table S1 shows the associations of adolescence dairy products intake with cIMT in early adulthood, it was observed that between dairy products, only the intake of cream could increase cIMT in early adults after 10 years (OR 0.245; 95% CI 0.001, 0.009; P: 0.009). Moreover, Additional file 2: Table S2 displays the associations of adolescence dairy products intake with components of MetS in early adulthood. In this regard, higher skim milk consumption increased TG (OR 0.189; 95% CI 0.000, 0.001; P: 0.038) and DBP (OR 0.212; 95% CI 0.002, 0.031; P: 0.029) whereas plain yoghurt (OR − 0.193; 95% CI − 0.048, − 0.001; P: 0.039). In addition, the intake of plain ice cream had reverse association with WC after full adjustment with confounding factors (OR − 0.166; 95% CI − 0.250, − 0.024; P: 0.018).

## Discussion

In the current study, we found no association of adolescence total calcium, dairy and non-dairy calcium intake with early adulthood cIMT. However, adolescents with the intake of cream had increased cIMT in their early adulthood. Furthermore, no higher MetS in the early adulthood was observed in the adolescents with higher consumption of dairy and non-dairy calcium. Moreover, skim milk, non-dairy and total calcium intake, but not other dairy products, could increase TG. The intake of plain ice cream showed reverse association with WC after 10-year follow-up in young adults.

There are some studies examining the calcium and dairy products intake in childhood and its association with cardiovascular risk factors in adulthood [10, 12, 17, 32–35]. Similar to our findings, the research conducted by Wu et al. in 3–18 years old participants concluded that there were not any linear and nonlinear relationships between long term dietary calcium intake (mean: 1020 mg/d in women and 1283 mg/d in men) with cIMT, PWV, stiffness index, carotid artery compliance, and Young's elastic modulus [12]. In terms of dairy products, although we could observe the relationship between long-term intake of cream and elevated cIMT, another research found no significant linkage between childhood dairy intake with adulthood coronary heart disease (CHD) and stroke mortality, while they observed that 400 mg/d calcium consumption had an association with decreased 40–60% stroke mortality [32]. Interestingly, a U-shaped association in 20-year longitudinal study in China was seen between calcium intake of adolescents and hypertension in adults. Meaning that lower and higher intake of calcium in adolescence can cause high blood pressure in later life [33]. We demonstrated that the consumption of plain yoghurt can decrease SBP. Concerning another CVD risk factor, an evidence found no relationship between long-term calcium intake (mean:1019 mg/d in female, 1270 mg/d in males) in youth with risk of type 2 diabetes in adulthood [34]. Whereas, high school adolescents with dairy product intake demonstrated 38% lower risk of type 2 diabetes in middle-aged women [35]. According to some other surveys claimed that there might be detrimental impact of calcium supplementation on CVD in adults [10, 17], all above mentioned studies did not indicate the harmful influence after long-term consumption of calcium and dairy products, whereas, long-term intake of cream probably could increase cIMT as well as the consumptions of dairy and non-dairy products might raise the components of MetS including TG and DBP as the current study found. Thus far, because there are conflicting conclusions between these evidence, and there has not been illustrated the effect of long-term intake of calcium supplements on cardiovascular causes yet [36], it could be due to the differences between the source of calcium intake.

On the other hand, there are some cross-sectional studies related to the linkage of calcium and dairy consumption with CVD risk factors needing to be mentioned. Although, we observed no long-term relationship of dairy and non-dairy calcium intake with MetS in our findings, we have previously observed that low-fat dairy products reduced risk of MetS in children and adolescents of the same population (TLGS) [37]. Moreover, in another study, adults showed that higher calcium intake (mean calcium intake: 461 mg/d in men and 426 mg/d in women) could diminish the risk of CVD only in women [38]. With regard to food pattern changes, the Bogalusa Heart Study indicated that from childhood to adulthood, there was a decrease in consumption of milk, especially in males [39]. It seems that adults are more likely to adhere to unhealthy dietary patterns than children as we also observed that adolescents had higher intake of dairy and total calcium in comparison with adults. Besides, in adults, it was observed that the intake of low-fat dairy had association with reduced PWV and cIMT [40]. Although, cheese and milk consumption did not have the relationship with cIMT, 100 g yoghurt/d could lower cIMT in elderly women [41]. In contrast, it was seen that calcium consumption with supplements resulted in escalated cIMT in postmenopausal women with dyslipidemia [11]. Overall, observing cross-sectional evidence seemed like that adulthood intake of calcium from dairy sources can reduce cIMT and other CVD risks.

The reason that there has not been the link between calcium intake and cardiovascular risks after a long time might be the independent role of circulating calcium levels from the sources of supplements, although it was claimed that circulating calcium can lead to artery calcification and clotting [10]. In the current study, we evaluated this relationship in young adults which may be early to observe plaques and therefore increased cIMT, notwithstanding elevated cIMT and performing plaques can happen in early stage of life [42]. Hence, calcium deposition has not been caused by the intake of dietary calcium and dairy products. It is worth mentioning that calcium begins its protective and lifelong role in cardiovascular risk factors from early in life so that it is necessary to consider diet from childhood to prevent them from chronic diseases in adulthood. Another hypothesis related to the influence of dairy products on artery structure is about lipid content of this product, in particular saturated fatty acid (SFA) having association with many negative health effects which may cause increased LDL levels, and thus an increased risk of CVD [43]. Regarding this, we could observe the high consumption of cream consisting of SFA associated with elevated cIMT [44]. However, findings have indicated that the link between SFA and CVD could not be considered evident than previously assumed due to the different combination of foods in saturated and unsaturated fatty acids, as well as contribution of significant quantities of other nutrients such as potassium and magnesium that may alter CVD risk [45].

The present study had some strength needing to be mentioned. First, it had a cohort design in which we observed the tracking intake of calcium and dairy products from adolescence to adulthood. Secondly, it was considered both cIMT and MetS which are strong risk factors for CVD since childhood and adolescence. Also, it is prominent to interpret the study in the light of limitations. We could only regard young adults as their cIMT were measured and this period of time is early to observe increased cIMT. Also, 10 years of follow-up was a short-term for observing the changes of artery structure. We did not consider other source of calcium as supplements that my change the results. Furthermore, changes the diet and food items during 10 years should have considered as it might affect the outcomes. Another limitation was that there are some confounders that we did not consider in the current study. As the cohort study used 168-item FFQ, we could extract only calcium products included in this questionnaire, and we did not consider all calcium-rich foods in the current study. So that this was also another limitation of this study. We suggested that further studies need to examine these relationships in a larger sample size.

## Conclusions

Adolescence calcium and dairy products could not lead to increased cIMT and MetS in early adulthood. Although, it is suggested that cream should be consumed with caution. Moreover, long-term intake of total calcium, non-dairy, and skim milk could increase TG and DBP in young adults.

## Supplementary Information

**Additional file 1**. Flow Chart of Study Participants.

**Additional file 2**. Associations of adolescence each dairy product intake with cIMT and MetS in early adulthood.

## Data Availability

The datasets used and/or analysed during the current study are available from the corresponding author on reasonable request.

## Abbreviations

BMI: Body mass index
CVD: Cardiovascular disease
cIMT: Carotid intima media thickness
CHD: Coronary heart disease
DBP: Diastolic blood pressure
FBS: Fasting blood sugar
FCT: Food composition table
FFQ: Food Frequency Questionnaire
HDL: High-density lipoprotein
ICC: Interclass correlation coefficient
JIS: Joint interim statement
LCCA: Left common carotid artery
LRC: Lipid research clinics
LDL: Low-density lipoprotein
MetS: Metabolic syndrome
MAQ: Modifiable Activity Questionnaire
PWV: Pulse wave velocity
SFA: Saturated fatty acid
SBP: Systolic blood pressure
TLGS: Tehran lipid and glucose study
TG: Triglyceride
USDA: United States Department of Agriculture
WC: Waist circumference
