# Supplementary material for: The association of long-term calcium and dairy products intake in adolescence with carotid intima media thickness and metabolic syndrome in early adulthood: Tehran Lipid and Glucose Study
Source: Nutr Metab (Lond). 2023 Apr 3;20:21. doi: 10.1186/s12986-023-00725-4 (PMC10069023; doi:10.1186/s12986-023-00725-4)
Supplement: Supplementary file 1 — Additional file 1. Flow Chart of Study Participants. [file 12986_2023_725_MOESM1_ESM.docx]

The third phase (n=12523)

*Third phase*: 12-19 (n=1546)

*Sixth phase*: over 20 (n=1024)

Over- and under-reported energy intake (±3 SD) data (n=1)

Having FFQ in the third phase (n=274)

***Exclusion***

use of any medications and supplements

Subjects for final analysis (n=217)

Underweight at baseline (n=33)

Having diseases such as high blood pressure, hyperglycemia, and high lipid profile (n=23)
