# Supplementary material for: The association of long-term calcium and dairy products intake in adolescence with carotid intima media thickness and metabolic syndrome in early adulthood: Tehran Lipid and Glucose Study
Source: Nutr Metab (Lond). 2023 Apr 3;20:21. doi: 10.1186/s12986-023-00725-4 (PMC10069023; doi:10.1186/s12986-023-00725-4)
Supplement: Supplementary file 2 — Additional file 2. Associations of adolescence each dairy product intake with cIMT and MetS in early adulthood. [file 12986_2023_725_MOESM2_ESM.docx]

| **Supplement 1**. Associations of adolescence dairy products intake with cIMT in early adulthood. (n=217) | |
| --- | --- |
| **Food Items** | **cIMT**  **SC (β)** **P-value** |
| Skim milk (mg)  Model 1  Model 2 | \| 0.007 \| 0.940 \| \| --- \| --- \| \| 0.004 \| 0.967 \| |
| Whole milk (mg)  Model 1  Model 2 | \| -0.036 \| 0.677 \| \| --- \| --- \| \| -0.049 \| 0.597 \| |
| Chocolate milk (mg)  Model 1  Model 2 | \| -0.030 \| 0.735 \| \| --- \| --- \| \| -0.015 \| 0.880 \| |
| Plain yoghurt (mg)  Model 1  Model 2 | \| -0.031 \| 0.727 \| \| --- \| --- \| \| -0.056 \| 0.573 \| |
| Whole yoghurt (mg)  Model 1  Model 2 | \| 0.021 \| 0.814 \| \| --- \| --- \| \| -0.009 \| 0.923 \| |
| Cheese (mg)  Model 1  Model 2 | \| 0.048 \| 0.579 \| \| --- \| --- \| \| 0.042 \| 0.645 \| |
| Creamy cheese (mg)  Model 1  Model 2 | \| 0.010 \| 0.904 \| \| --- \| --- \| \| 0.087 \| 0.367 \| |
| Dough (mg)  Model 1  Model 2 | \| -0.010 \| 0.910 \| \| --- \| --- \| \| -0.026 \| 0.784 \| |
| Cream (mg)  Model 1  Model 2 | \| 0.221 \| **0.011** \| \| --- \| --- \| \| 0.245 \| **0.009** \| |
| Traditional ice cream (mg)  Model 1  Model 2 | \| 0.040 \| 0.723 \| \| --- \| --- \| \| -0.011 \| 0.926 \| |
| Plain ice creams (mg)  Model 1  Model 2 | \| 0.080 \| 0.355 \| \| --- \| --- \| \| 0.050 \| 0.594 \| |
| Kashk (mg)  Model 1  Model 2 | \| -0.091 \| 0.297 \| \| --- \| --- \| \| -0.094 \| 0.287 \| |
| Abbreviations: cIMT, carotid intima media thickness; SC, standardized coefficients.  Model 1. Adjusted for age, sex, body mass index, and physical activity.  Model 2. Adjusted for Model 1 and energy intake, smoking, family history of cardiovascular disease.  Using Linear regression test.  Standardized coefficients (β).  P-value <0.05 significant. | |

| **Supplement 2**. Associations (β, 95% CI) of adolescence dairy products intake with the components of MetS in early adulthood. (n=217) | | | | | | |
| --- | --- | --- | --- | --- | --- | --- |
| **Food Items** | **TG**  **SC (β)** **P-value** | **WC**  **SC (β)** **P-value** | **FBS**  **SC (β)** **P-value** | **SBP**  **SC (β)** **P-value** | **DBP**  **SC (β)** **P-value** | **HDL**  **SC (β)** **P-value** |
| Skim milk (mg)  Model 1  Model 2 | \| 0.209 \| **0.021** \| \| --- \| --- \| \| 0.189 \| **0.038** \| | \| -0.019 \| 0.792 \| \| --- \| --- \| \| -0.051 \| 0.467 \| | \| 0.035 \| 0.717 \| \| --- \| --- \| \| 0.020 \| 0.829 \| | \| -0.036 \| 0.704 \| \| --- \| --- \| \| -0.013 \| 0.889 \| | \| 0.207 \| **0.031** \| \| --- \| --- \| \| 0.212 \| **0.029** \| | \| -0.171 \| 0.062 \| \| --- \| --- \| \| -0.139 \| 0.117 \| |
| Whole milk (mg)  Model 1  Model 2 | \| 0.029 \| 0.741 \| \| --- \| --- \| \| 0.082 \| 0.390 \| | \| 0.053 \| 0.442 \| \| --- \| --- \| \| 0.036 \| 0.622 \| | \| 0.143 \| 0.122 \| \| --- \| --- \| \| 0.132 \| 0.178 \| | \| -0.113 \| 0.215 \| \| --- \| --- \| \| -0.101 \| 0.305 \| | \| 0.039 \| 0.678 \| \| --- \| --- \| \| 0.010 \| 0.919 \| | \| -0.025 \| 0.780 \| \| --- \| --- \| \| 0.050 \| 0.594 \| |
| Chocolate milk (mg)  Model 1  Model 2 | \| -0.053 \| 0.548 \| \| --- \| --- \| \| 0.001 \| 0.991 \| | \| 0.099 \| 0.151 \| \| --- \| --- \| \| 0.088 \| 0.238 \| | \| 0.045 \| 0.627 \| \| --- \| --- \| \| 0.029 \| 0.774 \| | \| -0.020 \| 0.826 \| \| --- \| --- \| \| 0.051 \| 0.610 \| | \| 0.169 \| 0.071 \| \| --- \| --- \| \| 0.198 \| 0.056 \| | \| -0.073 \| 0.420 \| \| --- \| --- \| \| -0.002 \| 0.981 \| |
| Plain yoghurt (mg)  Model 1  Model 2 | \| -0.008 \| 0.932 \| \| --- \| --- \| \| 0.040 \| 0.685 \| | \| 0.002 \| 0.980 \| \| --- \| --- \| \| -0.034 \| 0.655 \| | \| -0.019 \| 0.844 \| \| --- \| --- \| \| -0.056 \| 0.576 \| | \| -0.193 \| **0.039** \| \| --- \| --- \| \| -0.159 \| 0.113 \| | \| -0.041 \| 0.675 \| \| --- \| --- \| \| -0.058 \| 0.579 \| | \| -0.094 \| 0.311 \| \| --- \| --- \| \| -0.020 \| 0.830 \| |
| Whole yoghurt (mg)  Model 1  Model 2 | \| -0.016 \| 0.861 \| \| --- \| --- \| \| 0.037 \| 0.703 \| | \| 0.120 \| 0.082 \| \| --- \| --- \| \| 0.109 \| 0.136 \| | \| 0.043 \| 0.644 \| \| --- \| --- \| \| -0.017 \| 0.864 \| | \| -0.038 \| 0.679 \| \| --- \| --- \| \| -0.009 \| 0.925 \| | \| 0.118 \| 0.213 \| \| --- \| --- \| \| 0.097 \| 0.348 \| | \| -0.069 \| 0.448 \| \| --- \| --- \| \| 0.031 \| 0.744 \| |
| Cheese (mg)  Model 1  Model 2 | \| -0.069 \| 0.436 \| \| --- \| --- \| \| -0.037 \| 0.684 \| | \| 0.093 \| 0.174 \| \| --- \| --- \| \| 0.089 \| 0.199 \| | \| 0.051 \| 0.581 \| \| --- \| --- \| \| 0.028 \| 0.766 \| | \| -0.040 \| 0.660 \| \| --- \| --- \| \| -0.029 \| 0.754 \| | \| 0.070 \| 0.455 \| \| --- \| --- \| \| 0.057 \| 0.597 \| | s   \| -0.035 \| 0.695 \| \| --- \| --- \| \| 0.010 \| 0.910 \| |
| Creamy cheese (mg)  Model 1  Model 2 | \| -0.071 \| 0.425 \| \| --- \| --- \| \| -0.060 \| 0.514 \| | \| 0.003 \| 0.964 \| \| --- \| --- \| \| 0.001 \| 0.992 \| | \| -0.042 \| 0.649 \| \| --- \| --- \| \| -0.009 \| 0.928 \| | \| -0.037 \| 0.683 \| \| --- \| --- \| \| 0.010 \| 0.914 \| | \| -0.020 \| 0.829 \| \| --- \| --- \| \| 0.019 \| 0.847 \| | \| 0.157 \| 0.080 \| \| --- \| --- \| \| 0.153 \| 0.085 \| |
| Dough (mg)  Model 1  Model 2 | \| 0.037 \| 0.687 \| \| --- \| --- \| \| 0.096 \| 0.328 \| | \| 0.032 \| 0.652 \| \| --- \| --- \| \| 0.004 \| 0.961 \| | \| -0.053 \| 0.578 \| \| --- \| --- \| \| -0.105 \| 0.296 \| | \| -0.147 \| 0.114 \| \| --- \| --- \| \| -0.123 \| 0.218 \| | \| -0.016 \| 0.870 \| \| --- \| --- \| \| -0.046 \| 0.662 \| | \| -0.035 \| 0.707 \| \| --- \| --- \| \| 0.054 \| 0.569 \| |
| Cream (mg)  Model 1  Model 2 | \| -0.093 \| 0.291 \| \| --- \| --- \| \| -0.049 \| 0.601 \| | \| 0.019 \| 0.786 \| \| --- \| --- \| \| 0.007 \| 0.925 \| | \| 0.038 \| 0.679 \| \| --- \| --- \| \| 0.029 \| 0.765 \| | \| -0.089 \| 0.327 \| \| --- \| --- \| \| -0.064 \| 0.506 \| | \| 0.061 \| 0.517 \| \| --- \| --- \| \| 0.052 \| 0.606 \| | \| -0.013 \| 0.885 \| \| --- \| --- \| \| 0.039 \| 0.670 \| |
| Traditional ice cream (mg)  Model 1  Model 2 | \| -0.070 \| 0.509 \| \| --- \| --- \| \| -0.041 \| 0.708 \| | \| -0.021 \| 0.809 \| \| --- \| --- \| \| -0.039 \| 0.663 \| | \| 0.132 \| 0.258 \| \| --- \| --- \| \| 0.112 \| 0.342 \| | \| 0.025 \| 0.826 \| \| --- \| --- \| \| 0.035 \| 0.765 \| | \| 0.120 \| 0.293 \| \| --- \| --- \| \| 0.093 \| 0.430 \| | \| -0.079 \| 0.457 \| \| --- \| --- \| \| -0.034 \| 0.746 \| |
| Plain ice cream (mg)  Model 1  Model 2 | \| -0.127 \| 0.155 \| \| --- \| --- \| \| -0.164 \| 0.077 \| | \| -0.097 \| 0.163 \| \| --- \| --- \| \| -0.166 \| **0.018** \| | \| 0.037 \| 0.693 \| \| --- \| --- \| \| -0.025 \| 0.797 \| | \| -0.089 \| 0.333 \| \| --- \| --- \| \| -0.048 \| 0.612 \| | \| -0.011 \| 0.911 \| \| --- \| --- \| \| -0.029 \| 0.769 \| | \| 0.000 \| 0.997 \| \| --- \| --- \| \| 0.115 \| 0.202 \| |
| Kashk (mg) Model 1  Model 2 | \| -0.022 \| 0.807 \| \| --- \| --- \| \| 0.010 \| 0.915 \| | \| -0.092 \| 0.181 \| \| --- \| --- \| \| -0.089 \| 0.193 \| | \| -0.007 \| 0.941 \| \| --- \| --- \| \| -0.002 \| 0.987 \| | \| 0.023 \| 0.803 \| \| --- \| --- \| \| 0.030 \| 0.749 \| | \| -0.096 \| 0.307 \| \| --- \| --- \| \| 0.092 \| 0.338 \| | \| 0.031 \| 0.727 \| \| --- \| --- \| \| 0.035 \| 0.690 \| |
| Abbreviations: DBP, diastolic blood pressure; FBS, fasting blood sugar; HDL-C, high density lipoprotein-cholesterol; MetS, metabolic syndrome; SBP, systolic blood pressure; SC, standardized coefficients; TG, triglyceride; WC, waist circumference.  Model 1. Adjusted for age, sex, body mass index, and physical activity.  Model 2. Adjusted for Model 1 and energy intake, smoking, family history of cardiovascular disease.  Using Linear regression test.  P-value <0.05 significant. | | | | | | |
